# Sclerosing mesenteritis due to *Mycobacterium genavense* infection: A case report

**DOI:** 10.1097/MD.0000000000030351

**Published:** 2022-09-09

**Authors:** Francisca Artigues Serra, Mercedes García-Gasalla, Antoni Campins, Miguel González de Cabo, Rafael Morales, Rebecca Rowena Peña, María Carmen Gallegos, Melchor Riera

**Affiliations:** a Infectious Diseases Unit, Internal Medicine Department, Hospital Universitari Son Espases-IdISBa, Palma, Spain; b Radiology Department, Hospital Universitari Son Llàtzer-IdIsBa, Palma, Spain; c Peritoneal Surface Malignancies Unit, General and Digestive Surgery Department, Hospital Universitari Son Espases-IdISba, Palma, Spain; d Microbiology Department, Hospital Universitari Son Llàtzer-IdIsBa, Palma, Spain.

**Keywords:** case report, Mycobacteria, sclerosing mesenteritis, surgical treatment

## Abstract

Since its first identification in the early 1990s, *Mycobacterium genavense* has been considered and opportunistic pathogen. It mainly causes gastrointestinal symptoms, but also disseminated infections in severely immunosuppressed patients. Sclerosing mesenteritis is a long-term complication with high morbidity and mortality. As it is a rare condition, there are no specific guidelines for its management. We report a challenging case of persistent *M. genavense* infection, and propose surgery as an alternative treatment strategy.

**Patient concerns:**

A 38-year-old Caucasian man presented to the emergency room with fever, abdominal pain, and night sweats for 3 months. HIV screening revealed a previously unknown HIV-1 infection, with a CD4 cell count of 216 cell/µL and viral load of 361.000 copies/mL at diagnosis. A body CT-scan showed mild splenomegaly as well as mesenteric and retroperitoneal enlarged lymph nodes. Fine needle aspiration revealed the presence of acid-fast bacilli, but mycobacterial cultures were negative. In the second sample, 16S RNA sequencing yielded a diagnosis of *M. genavense* infection. Despite 2 years of corticosteroids and antimycobacterial treatment excluding rifampicin due to a severe cutaneous reaction, there was no clinical improvement and an increase in the mesenteric lymph node size was observed, with a sclerosing transformation of the mesentery. A surgical approach was proposed to release small bowel loops and to remove fibrin. A second surgery was required due to an acute peritonitis ought to yeyunal segmental isquemia and perforation. Finally, the patient evolved favorably, and antimycobacterial drugs were suspended without relapse.

**Lessons.:**

Despite a prolonged multidrug strategy, some patients develop persistent *M. genavense* infection. Once sclerosing mesenteritis is established, clinicians have few treatment options. Surgery should be considered in patients with sclerosing mesenteritis or bowel obstruction. The combination of medical and surgical treatment could be a potential cure for these patients.

## 1. Introduction

*Mycobacterium genavense* is a nontuberculous mycobacterium first described in 1990 by Hirschel et. al.^[1]^ in a patient with acquired immunodeficiency syndrome (AIDS) who presented with diarrhea, weight loss and fever. Acid-fast bacilli were found in samples of intestine, liver, spleen, and lymph nodes, among other tissues. However, it was not identified until 2 years later by Bottger et. al.^[2]^ using 16S ribosomal RNA gene sequencing and amplification. They reported a series of 18 HIV-seropositive patients with similar abdominal symptoms, suggesting the identification of a new mycobacteria species. Nowadays it is known that *M. genavense* belongs to the *Mycobacterium simiae complex* group.

*M. genavense* is considered a ubiquitous opportunistic pathogen. An important risk factor is impaired cellular immune response, as seen in HIV-infected patients with low CD4 cell counts but also in patients after solid-organ or hematopoietic stem cell transplantation and in those receiving immunosuppressive therapy.^[3,4]^ Late identification is common due to difficulties in culturing the microorganism.^[5]^ Most information comes from case reports and small series, which reflects the rarity of this infection. Sclerosing mesenteritis has been described as a potential pathogen-specific syndrome in some patients, mimicking lymphoma or peritoneal carcinomatosis.^[6,7]^ It is characterized by a diffuse, localized or multinodular thickening of the mesentery, with chronic inflammation and fibrosis, causing thrombotic events and great morbidity.^[6,8]^ As it is a rare condition, experience about its optimal management is scarce. Here we present a case of persisting *M. genavense* infection in an HIV-infected patient who developed sclerosing mesenteritis and in whom a combination of medical and surgical treatment was proposed.

## 2. Case presentation

A 38-year-old Caucasian man presented to the Emergency Department in February 2019 with 3 months of generalized malaise, abdominal pain, diarrhea, fever up to 38ºC and night sweats. He had a history of major depressive disorder and 2 cervical disc herniations which were treated by microdiscectomy 1 year previously. He had been living in an urban area in Spain, without recent travel. On physical examination, the patient was hemodynamically stable. Neurological examination and cardiopulmonary auscultation were normal, tenderness in the epigastric and left hypochondrium regions was found, with no masses nor hepatosplenomegaly. Painful small (<1cm) lymph nodes were detected in the left inguinal and left cervical zones.

The laboratory study revealed mild anemia and slightly elevated C-reactive protein. White blood count, platelets, coagulation, renal function, albumin, and liver function tests were normal. Blood and stool cultures were negative. HIV screening was positive with ELISA and Western blot test for infection with HIV-1. The patient did not know this information previously. CD4 + T-lymphocyte count was 216 cells/uL (8%) and HIV viral load was 361.000 copies/mL. Interferon-gamma release assay (IGRA) and serological evaluation for Rubella, hepatitis virus, Epstein-Barr virus, Erythrovirus B19, *Treponema pallidum*, *Coxiella burnetii*, *Rickettsia conorii*, *Bartonella henselae* and *Bartonella quintana* were negative.

**Figure 1.**
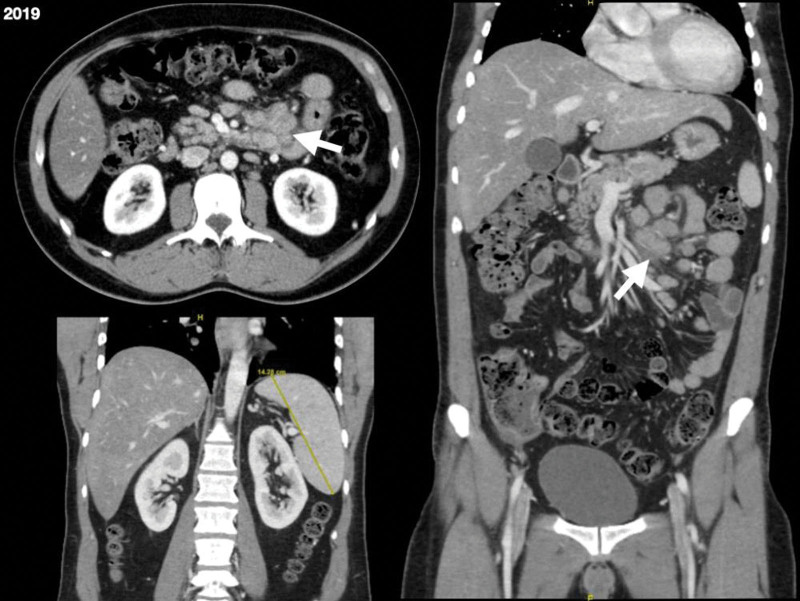
Images of the CT-scan performed in February 2019. White arrows show mesenteric lymph node enlargement. Mild splenomegaly (14 cm) is also indicated.

The abdominal ultrasound showed moderate hepatic steatosis and mild splenomegaly (14cm). A body CT-scan was also performed, revealing mesenteric and retroperitoneal lymph node enlargement with unspecific edema of the jejunum wall (Fig. 1). Endoscopic examination was normal. Acid-fast bacilli staining of the lymph-node aspiration specimen yielded positive results; however, polymerase chain reaction (PCR) analysis for *Mycobacterium tuberculosis* was negative. Ziehl- Nielsen staining of stool samples was also positive. Abdominal pain was controlled and empirical antimycobacterial treatment was started with isoniazid, rifampicin, pyrazinamide, ethambutol, and azithromycin while waiting for the mycobacterial cultures. Antiretroviral therapy (ART) was delayed.

Two weeks later, the patient was readmitted with fever, worsening abdominal pain, nausea and anorexia. A bulky lymph node enlargement was observed and he was diagnosed with paradoxical immune reconstitution inflammatory syndrome and high-dose corticosteroids were started. Prophylactic cotrimoxazole was also introduced because of decreasing levels of CD4 cells (117 cells/uL, 7%). The patient evolved favorably and was discharged with low doses of prednisone.

In April 2019 ART was started with raltegravir, tenofovir alafenamide and emtricitabine. CD4 nadir was 64 cells/uL, with undetectable viral load since ART. During the follow-up in the outpatient clinic, rifampicin and cotrimoxazole were discontinued due to Stevens-Johnson syndrome and he continued treatment with ethambutol, azithromycin, fluoroquinolones and low-dose corticosteroids therapy. Rifampicin desensitization was performed with unsuccessful results. No clinical nor radiological improvement was observed. Mycobacterial cultures were finally negative after 12 weeks of incubation, so a second abdominal lymph node sample was obtained by fine-needle aspiration and a 16S rRNA sequence analysis was performed, confirming the diagnosis of *Mycobacterium genavense* infection.

The patient’s ART was simplified to biktegravir, tenofovir alafenamide and emtricitabine. In January 2020 he was readmitted with severe abdominal pain, requiring high doses of corticosteroids and opioids. He was discharged with prednisone 60 mg/day, and an opioid-based regimen to control symptoms. In October 2020, ethambutol was stopped due to ocular toxicity, introducing clofazimine and amikacin in addition to azithromycin and levofloxacin.

**Figure 2.**
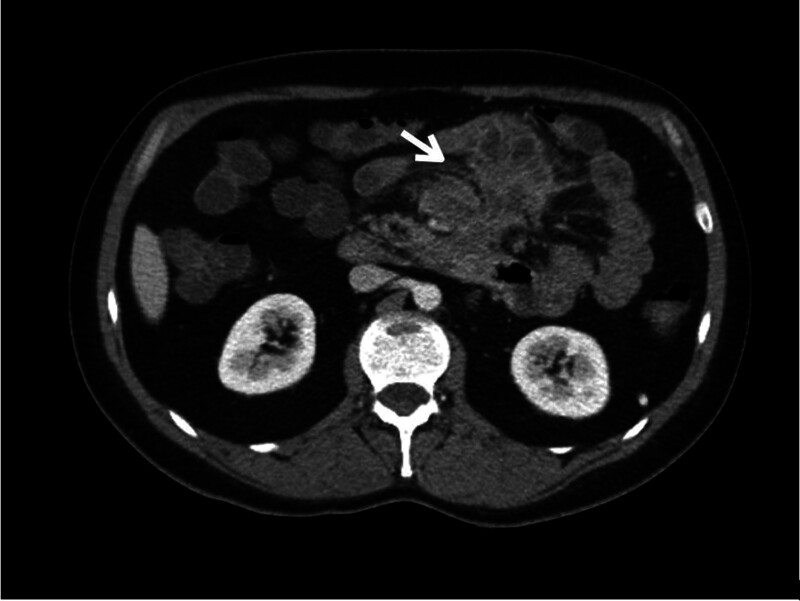
Images of the CT-scan performed in May 2021. White arrow shows mesenteric sclerosis.

**Figure 3.**
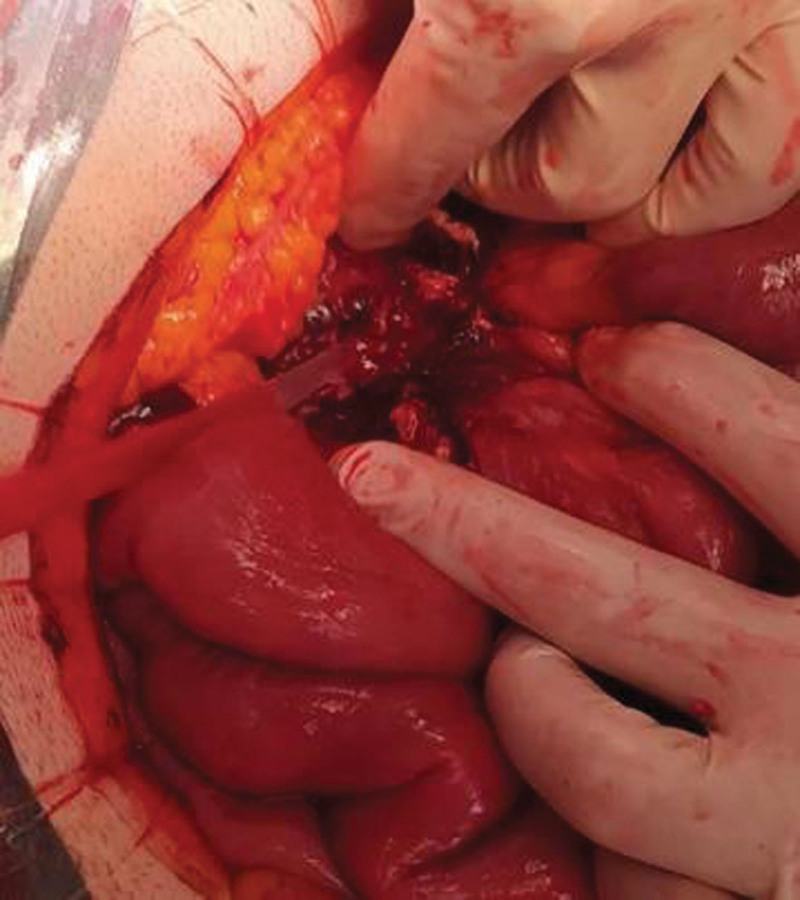
Intraoperative images showing retraction of the mesentery, fibrin and profuse inflammatory exudate.

In spite of the antimycobacterial treatment, no improvement was observed, and the patient was hospitalized with severe abdominal pain, anorexia, weight loss and daily fever. The CT-scan revealed sclerosing transformation of the mesentery with local fibrosis retracting jejunum and occluding the superior mesenteric vein (Fig. 2). High uptake of fluorodeoxyglucose (FDG) was observed in PET/CT imaging, suggesting an active, persistent infectious process with abscessification in the mesenteric root. The patient underwent laparotomy, which showed an extensive inflammatory retractile mass in the mesentery root, causing adhesions and partial obstruction of the bowel, with profuse inflammatory exudate (Fig. 3). Small bowel loops were released and fibrin and caseum were removed.

During the postoperative days acute peritonitis developed. The CT scan showed several peritoneal abscesses, so a second surgical intervention was necessary. An intestinal isquemic yeyunal segment with perforation was confirmed and a 35cm-long bowel resection was performed. Histologically, the submucosal connective tissue revealed severe chronic inflammation and fibrosis with necrotizing granulomas, but acid-fast bacilli were not identified. The patient recovered well. He was discharged with clofazimin, azitromicin, levofloxacin, and prednisone 10mg/24h.

**Figure 4.**
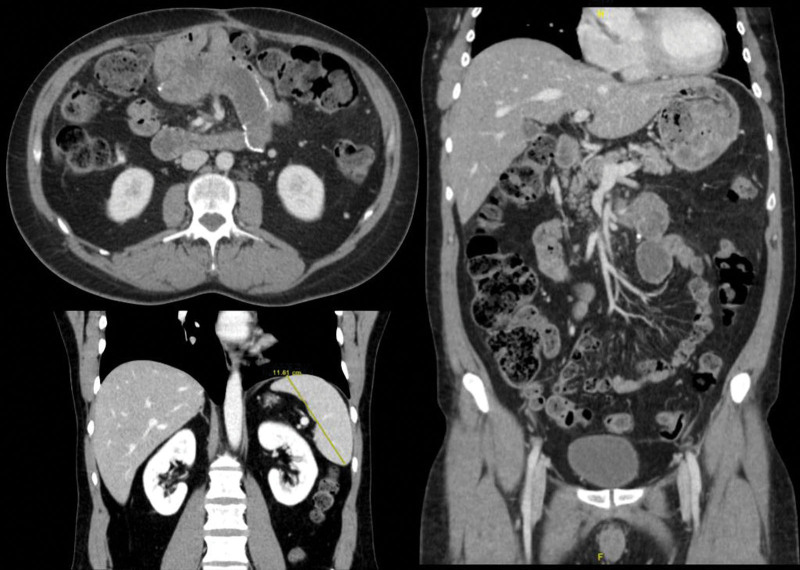
Images of the CT-scan performed in December 2021. It shows radiological improvement, without peritoneal abscesses nor mesenteric lymph node enlargement.

By December 2021, clinical and radiological improvement was evidenced (Fig. 4), and antimycobacterial treatment was stopped. CD4 cell count was 250 cells/uL and HIV viral load was < 20 copies/mL. No relapses have been reported since then, with a subsequent improvement in the patient`s quality of life.

## 3. Discussion

Among HIV-seropositive patients, disseminated infection by nontuberculous mycobacteria is considered an AIDS-defining condition.^[9]^ *Mycobacterium genavense* belongs to this group of microorganisms, affecting people with severe immunosuppression. The level of immune dysfunction is related to CD4 cell count, which is usually lower than 100 cells/mm^3^ at diagnosis.^[2]^ Since the introduction of ART, the survival in this group of patients has significantly improved, with an increase of CD4 cells.^[10]^ In our patient, the initial CD4 cell count was surprisingly higher (216 cell/uL), although CD4 nadir during this period of time was 64 cells/uL. CD4 cell count was persistently low despite ART and suppressed viremia. This finding is probably due to the persistent mycobacterial infection and a chronic underlying inflammatory response.

The disease pattern suggests an intestinal rather than a respiratory infection pathway.^[11]^ Clinical features of *M. genavense* infection are similar to MAC, including asthenia, weight loss, fever, splenomegaly and diarrhea.^[4]^ However, abdominal pain is more common in disseminated infection by *M. genavense* than by MAC,^[6,11]^ which is probably related to the amount of acid-fast bacilli in the intestinal mucosa.^[12]^

The diagnosis is challenging due to the fastidious growth requirements.^[3,5]^ One of the best options to culture *M. genavense* is with solid media supplemented with blood and charcoal and acidified to pH 6.2 ± 0.2.^[3]^ Recommended incubation period is 8–12 weeks.^[5]^ Therefore, the diagnosis may be missed if it is not suspected. As in the present case, molecular techniques are necessary for an earlier identification.^[11,13]^

As *M. genavense* does not grow in standard media, data on drug susceptibility is difficult to obtain. According to published data, most isolates are susceptible in vitro to fluoroquinolones, clofazimine, and amikacin, although susceptibility testing is not standardized; treatment success has been described with macrolides and ethambutol in addition to rifampicin, amikacin, or a fluoroquinolone.^[5,14]^ Isoniazid is an option with limited evidence.^[5]^ Clofazimine has shown excellent in vitro susceptibility results to nontuberculosis mycobacteria.^[15,16]^ It is a well-tolerated drug and could be an alternative to rifamycins.^[17]^ The IDSA guidelines and a recent consensus guideline recommend multidrug therapies with at least 3 drugs, including macrolides plus rifampicin, ethambutol, moxifloxacin, clofazimine or amikacin.^[14,18]^ Antibiotics are usually maintained for prolonged periods of time, at least 12–18 months, even lifelong if immune restoration is not achieved.^[5,14]^ In the French series by Charles et. al., corticosteroid therapy was also used to control the immune reconstitution inflammatory syndrome or abdominal pain and was not associated with worse results.^[4]^ In our patient, antimycobacterial treatment was challenging because of multiple adverse drug reactions, with unsuccessful rifampicin desensitization.

Sclerosing mesenteritis was first described in 1924.^[19–21]^ It is considered a rare condition (prevalence < 1%) characterized by chronic nonspecific inflammation, fat necrosis and fibrosis of the mesentery.^[21]^ The etiopathogenesis of sclerosing mesenteritis remains unknown, although infections have been proposed as an underlying mechanism.^[19–21]^ Treatment should be guided by suspected etiology.^[19]^ Few cases of secondary sclerosing mesenteritis due to *M. genavense* infection have been published.^[6–8,13]^

Persistent and relapsing infections have been described in the literature, sometimes related to short duration of antimycobacterial therapy.^[13]^ Our patient did not show signs of improvement after almost 2 years of medical treatment, presenting sclerosing mesenteritis as a late complication. Corticosteroids and tamoxifen have been proposed as a first-line therapy in idiopathic cases of sclerosing mesenteritis.^[19]^ However, this combination treatment was used in 1 patient with secondary sclerosing mesenteritis due to *M. genavense* infection with poor results.^[8]^

To the best of our knowledge, this is the first reported case of *M. genavense* infection successfully treated by surgery. Intestinal *M. tuberculosis* infections complicated with perforation or obstruction have already been surgically treated, essentially in an emergency situation, but surgical management of mesenteric lymphadenitis and caseum has rarely been described.^[22,23]^ We propose surgical debridement to reduce mycobacterial load in infections which develop sclerosing mesenteritis with intestinal obstruction or abscessification, although every patient needs an individual assessment due to the potential risks of this procedure.

## 4. Conclusions

In conclusion, *M. genavense* is a rare opportunistic pathogen which mainly affects gastrointestinal tract. Late diagnosis is common due to unspecific clinical manifestations and challenging identification. Sclerosing mesenteritis has been described as a potential complication, causing high morbidity and mortality. Based on our results, combining medical and surgical treatment could emerge as a possible option for persistent infections.

## Author contributions

Conceptualization: Mercedes García-Gasalla.

Data curation: Francisca Artigues Serra and Mercedes García-Gasalla.

Supervision: Melchor Riera.

Writing—original draft: Francisca Artigues Serra.

Writing—review and editing: Mercedes García-Gasalla, Antoni Campins, Miguel González de Cabo, Rafael Morales, Rebecca Rowena Peña, María del Carmen Gallegos, Melchor Riera.
